# Standardization of SYBR Green-Based Real-Time PCR Through the Evaluation of Different Thresholds for Different Skin Layers: An Accuracy Study and Track of the Transmission Potential of Multibacillary and Paucibacillary Leprosy Patients

**DOI:** 10.3389/fmicb.2021.758222

**Published:** 2021-12-07

**Authors:** Lais Sevilha-Santos, Selma Regina Penha Silva Cerqueira, Ciro Martins Gomes

**Affiliations:** ^1^Programa de Pós-Graduação em Ciências Médicas, Faculdade de Medicina, Universidade de Brasília, Brasília, Brazil; ^2^Programa de Pós-Graduação em Medicina Tropcial, Faculdade de Medicina, Universidade de Brasília, Brasília, Brazil

**Keywords:** *Mycobacterium leprae*, diagnosis, real-time polymerase chain reaction, transmission, leprosy

## Abstract

The development of new molecular techniques is essential for the early diagnosis of leprosy. Studies in the field have failed to elucidate the performance of these tests in clinical practice. We aimed to design a new primer pair for the repetitive element (RLEP) target of *Mycobacterium leprae* and to test the accuracy of SYBR green-based real-time PCR through the evaluation of different thresholds for different skin layers. We also aimed to track the transmission potential of multibacillary and paucibacillary leprosy patients. The *in vitro* validation of our reaction resulted in a quantification limit of 0.03 bacilli. We then conducted a cross-sectional/cohort-based study of diagnostic accuracy. Patients were included, and skin samples were divided into four layers: epidermis, superior dermis, inferior dermis, and hypodermis. We also quantified *M. leprae* in nasal swabs of the included patients and compared the results to the number of household contacts also diagnosed with leprosy. One hundred patients with a clinical presentation compatible with leprosy were allocated to the leprosy or control group. Although the parasite load was greater in the superior and inferior dermis, *M. leprae* DNA was found in all skin layers. The best sensitivity was observed for the superior dermis using the presence of any quantifiable bacillus DNA as the threshold [sensitivity=59.26% (95% CI=45.97–71.32)]. In the epidermis, setting 1 quantifiable bacillus as the threshold resulted in 100% specificity (95% CI=92.29–100). The number of bacilli found in nasal swabs was not significantly related to the number of household contacts also diagnosed with leprosy. Paucibacillary patients tested positive only for bacillus fragments in nasal swabs but not for the entire bacilli. We can conclude that superficial biopsies might result in sensitivity loss, although different skin sample types will have little influence on the final accuracy. In contrast, threshold changes greatly influence these properties. Paucibacillary patients may not be a relevant source of disease transmission.

## Introduction

Leprosy is caused by *Mycobacterium leprae* and *Mycobacterium lepromatosis*. Untreated multibacillary patients are considered the main source of transmission, which is most likely to occur through the airways (Ministério da Saúde, 2016). The best approach for avoiding leprosy transmission is based on early diagnosis and treatment (Gurung et al., 2019). Leprosy case definition is based on clinical evaluation and the use of slit skin smears (SSSs) (World Health Organization, 2018). Endemic countries are facing the problem of delayed diagnosis, which is followed by the occurrence of disabilities (Rathod et al., 2020; World Health Organization, 2020). This phenomenon is attributed to the poor technical training of healthcare providers as a result of centuries of negligence (Limeira et al., 2013; Frade et al., 2017). Therefore, the development of accurate complementary techniques would help break the transmission chain.

According to recent systematic reviews of the literature (World Health Organization, 2018; Gurung et al., 2019; Torres et al., 2021), studies evaluating complementary techniques for the diagnosis of leprosy still suffer from methodological concerns. The performance of these tests in the field is still unknown, as most studies have sought to investigate laboratory properties. Most studies in the field focus on laboratory validation of molecular techniques, but clinical studies simulating application in practice are rare (Donoghue et al., 2001; Gurung et al., 2019). The presence of incomparable thresholds makes the precise analysis of existing techniques a considerable challenge (Gurung et al., 2019). In this context, the amplification of nucleic acids using polymerase chain reaction (PCR) presents great potential due to the greater sensitivity of this method than classical parasitological techniques (Turankar et al., 2015; Sundeep Chaitanya et al., 2016; Beissner et al., 2019). In practice, even tests that were initially considered very sensitive for the diagnosis of leprosy can suffer a considerable reduction in performance due to existing limitations in the process of patient selection, sample collection, test replication, and interpretation.

Different types of samples can be used for molecular diagnosis. The type of sample used can also influence the result of the examination. As a result of bacillary tropism, skin biopsies are the most frequently studied type of clinical specimen (Gurung et al., 2019). As shown in other diseases, such as leishmaniasis, an in-depth study of the pathogen’s kinetics in human skin would be of great use for the development of new diagnostic techniques and for commenting on the pathophysiology of leprosy (Sevilha-Santos et al., 2018). This study will also be important for generating recommendations regarding the best techniques for the diagnosis of leprosy and which changes have a greater impact on accuracy properties. In addition, the study of other sample types such as swabs from superior airways can be a useful tool for monitoring the transmission potential of multibacillary and paucibacillary leprosy (Araujo et al., 2016).

We aimed to design a new primer pair targeting the repetitive element (RLEP) of *M. leprae* and to test the accuracy of a multilevel analysis involving three thresholds, four different types of skin layers, and nasal swab specimens using SYBR green-based real-time PCR in a cross-sectional/cohort-based method. We also aimed to monitor any skin and upper airway signs as markers of leprosy transmission by comparing the results of skin and nasal swab sample quantification to the number of household contacts also diagnosed with leprosy.

## Materials and Methods

### Recruitment

This research strictly follows a previously defined research protocol and is in compliance with STARD 2015: An Updated List of Essential Items for Reporting Diagnostic Accuracy Studies and QUADAS-2: A Revised Tool for the Quality Assessment of Diagnostic Accuracy Studies (Whiting et al., 2006; Bossuyt et al., 2015). The first contains an updated list of essential items for reporting studies on diagnostic accuracy, and the second is a quality analysis tool that includes methods for better validation of the results.

Patients were consecutively recruited at the leprosy outpatient clinic of the University Hospital of Brasília, University of Brasília (UnB), Brasília, Brazil, from August 2018 to August 2020. This reference center is responsible for the differential diagnosis of leprosy in patients referred from primary and secondary healthcare institutions. The same board-certified dermatologist always performed recruitment, and the laboratory examinations were performed at the UnB Dermatomycology Laboratory by a specialized biomedicist. Both specialists were blinded to the patients’ condition (diagnosis of leprosy or any differential diagnosis) until the end of the laboratory evaluation period. The inclusion criteria consisted of (1) any patient referred to the leprosy outpatient clinic for differential diagnosis and (2) patients who agreed to participate in the research. We did not include (1) patients who did not sign the informed consent form, (2) patients belonged to indigenous communities, or (3) patients who were under eighteen years of age due to local ethical restrictions.

### Leprosy Case Definition (Composite Reference Standard)

All included patients were subjected to the same set of examinations and were posteriorly allocated to the leprosy or control group. We divided patients into case and control groups according to the application of leprosy case definitions as follows: case patients were the confirmed leprosy patients based on the World Health Organization (WHO) criteria and control patients were patients with other skin conditions initially confused with leprosy by the primary and secondary healthcare facilities.

The index test (real-time PCR) was not used for leprosy case definition to avoid bias in the results. A diagnosis of leprosy was made according to the WHO criteria, and classification was conducted according to the Ridley-Jopling (R&J) criteria, supported by SSS results (Ministério da Saúde, 2016). As the study was performed in a tertiary hospital, all included patients were evaluated by SSS collected from two earlobe sites, one elbow site, and one additional site represented by the edge of a skin lesion or by an additional elbow site in case no skin alteration was present.

### Sample Collection, Preparation for DNA Extraction, and DNA Extraction

**Figure 1:**
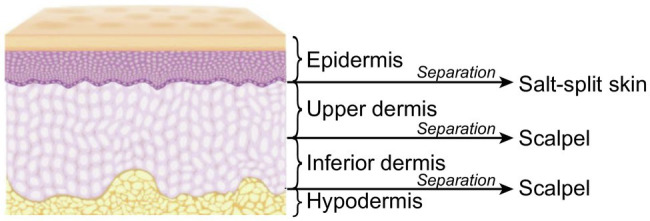
Schematic representation of the procedures used in the separation of skin layers.

The border of a suspected leprosy lesion was chosen as the site for sample collection, whereas for patients without well-delimited lesions showing only general skin infiltration (compatible with lepromatous leprosy), a fragment of the back of the right earlobe was collected. An incisional biopsy was performed after performing asepsis, antisepsis, and local anesthesia with a 2% lidocaine solution. The collected skin fragment was transversally divided into four parts: epidermis, upper dermis, inferior dermis, and hypodermis (Figure 1). The separation of the epidermis from the upper dermis was performed by the salt-split skin technique, which consists of incubating normal human skin in a 1.0M sodium chloride solution (1.0M NaCl) for 72–120h at −4°C (Rao et al., 2010; Sevilha-Santos et al., 2018). The division of the other layers was performed visually with a sharp and sterile scalpel. The differentiation of skin layers was easier in infiltrated sites according to the previous experience of the group (Sevilha-Santos et al., 2018). DNA extraction was performed in up to 24h after skin layer division and up to 96–120h after sample collection (samples were stored at −4°C to warrant similar conditions for all skin layers). We also collected nasal swab samples from the included patients as described elsewhere (Gomes et al., 2014). Swabs were stored at −4°C for up to 24h until DNA extraction. DNA extraction from skin and swab samples was performed using the PureLink Genomic DNA Mini Kit (Invitrogen, Thermo Fisher Scientific, Waltham, Massachusetts, United States) following the manufacturer’s instructions. This manufactured kit has previously defined protocols for the extraction of tissue and swab samples. DNA samples were stored at -80°C for up to 120h until real-time PCR reaction.

### Index Test

#### Real-Time PCR Primer Pair Design and *Mycobacterium leprae* Quantification Standardization

The primer pair specific for the *M. leprae* RLEP employed for real-time PCR was designed using the Primer designing tool (National Center for Biotechnology Information, Bethesda, Maryland, USA); the primer sequences were 5' – CTTGCACCATTTCTGCCGCT – 3' and 5' – TGCGCTAGAAGGTTGCCGTA – 3', which resulted in a 156bp fragment. We chose a primer pair with similar melting temperatures, 2°C degrees above 60°C (to use a single annealing/extension temperature), and a GC content lower than 50%.

A quantification standard curve was constructed using a cloned RLEP target found at locus 20ACBI4C_RLEP481, which resulted in a 481bp fragment. Cloning was performed by Thermo Fisher Scientific GENEART (Regensburg, Germany; Supplementary File 1). The number of copies in the stock solution resulting from the cloning of the 20ACBI4C_RLEP481 locus was equal to 9.63×10^12^. Assuming that 37 copies of this RLEP gene are equivalent to one bacillus, the number of bacilli was calculated as follows: $\frac{9.63x10^{12}}{37}$, so the stock solution contained 2.60×10^11^ bacilli. The standard curve was constructed with eight triplicate points with 1:10 serial dilutions from 7.8×10^7^ to 7.8 bacilli. Curve properties did not change when the positive control was diluted with good-quality human genomic DNA. We set the limit of quantification and detection to 0.03 bacilli, considering the minimum amount possibly detectable by the method (1/37)(R^2^ 0.99, efficiency 99.90, slope−3.32; Supplementary File 1).

The reactions were carried out in a QuantStudio 1 thermocycler (Applied Biosystems, Foster City, CA, United States) using PowerUp SYBR Green Master Mix (Applied Biosystems, Foster City, CA, United States) in a final volume of 15μl, containing 1X Universal SYBR Green PCR master mix (Applied Biosystems, Foster City, CA, United States), each primer at 10μM (Applied Biosystems, Foster City, CA, United States), 3μl DNA, and ultrapure water to the final volume. Amplification was performed with an initial step of 50°C for 2min and 95°C for 2min, followed by 45cycles of 95°C for 15s and 60°C for 1min. The melting curve was processed in increments of 0.3°C from 55°C to 95°C.

#### Quantitative Real-Time PCR for Human Beta Globulin

The chosen primer set was BGF 5' – GGCAGGTTGGTATCAAGGTTAC – 3' and BGR 5' – CCTAAGGGTGGGAAAATAGACC – 3', which are specific for human beta globulin. This endogenous target showed a better performance measured by standard curve efficiency than other targets tested in this validation process, including glyceraldehyde-3-phosphate dehydrogenase (Nygard et al., 2007) and C18X from the Homo sapiens isolate CHM13 chromosome 17 (Gomes et al., 2014). The reactions were carried out with the same thermocycler, final volume and mix used for the RLEP target. Amplification was performed with an initial step of 50°C for 2min and 95°C for 2min, followed by 45cycles of 95°C for 15s, 57°C for 15s and 72°C for 1min. The melting curve was processed in increments of 1.6°C from 60°C to 95°C. A five-point standard curve was constructed using 1:10 serial dilutions, starting with 20mg of skin tissue DNA equivalent per reaction (*R*^2^ 0.96, efficiency 100.16, slope−3.31).

#### Relative Quantification

Relative quantification for each of the skin layers was performed with the following calculation: $Relativequantity=\frac{M.lepraequantity\;}{Betaglobulinquantity}$ (Larionov et al., 2005). The results were expressed as the number of bacilli per mg of skin tissue.

#### Definition of Test Positivity and Diagnostic Thresholds

Real-time PCR results from skin samples were always compared to a standard curve performed in the same reaction plate. Positivity was defined as tests that presented amplification above the defined thresholds and with compatible melting curves. After the validation of the standard curve for *M. leprae* but prior to the processing of the samples, three thresholds were established for the samples to be considered positive. The positivity of a test was compared at the three following points: first – any quantifiable bacillus DNA; second – quantification result greater than or equal to 0.1 bacillus; and third – any quantification result greater than or equal to 1 bacillus. For all thresholds, positivity was also dependent on a compatible melting curve.

### Statistical Analysis

The number of household contacts of each index leprosy patient who was diagnosed with leprosy was also calculated. We also divided this number by the total number of contacts examined (relative number of sick household contacts), and this result was compared with the relative quantification results for all analyzed skin layers and the nasal swab quantification results. The Chi-square test or the exact version of this test was used to compare categorical variables. Student’s t test was used to evaluate the relationships of the data for the dependent samples. McNemar’s test was used for paired data. Sensitivity was calculated considering the percentage of positive results in patients with leprosy, and specificity was calculated according to the percentage of negative results in patients without leprosy. The accuracy value was obtained through the following calculation: $\frac{truepositive+truenegative\;}{totalpatients}$. We additionally evaluated the subgroups of paucibacillary and multibacillary patients separately. The construction of forest plots and summary receiver operating characteristic curves (SROCs) were performed in Review Manager (RevMan) version 5.3. (Copenhagen, Nordic Cochrane Centre, Cochrane Collaboration, 2014). Missing values were ignored in unpaired tests, and both groups that lost correspondents were excluded when applying paired tests. The R Studio program (Boston, United States) was used for the analyses. The significance threshold was set at a value of *p* <0.05 according to the 95% confidence interval (CI).

### Sample Size

In the sample size calculation, the following parameters were considered. First, we considered a sensitivity value of 78.5% (95% CI 61.9–89.2) and a specificity value of 89.3% (95% CI 61.4–97.8) for real-time PCR according to a previous meta-analysis (Andrade et al., 2021). We consider an initial sensitivity and specificity of 60% (H0), compatible with the lower limit of both confidence intervals described in the cited review (Andrade et al., 2021). The prevalence of patients diagnosed with leprosy within the service according to internal data is approximately 50%. We arbitrarily considered an improvement of 20% in the sensitivity and specificity of the index test considering the best sample and the best cutoff point (H1). Considering a power of 0.80 and a value of *p*<0.05, a minimum sample size of 45 patients per group (leprosy cases and controls) was reached for a total of 90 patients (Bujang and Adnan, 2016). An increase of 10% in this calculation was considered to supply the deleterious effects of the losses.

### Ethics Statement

This study was performed in accordance with the Declaration of Helsinki and was approved by the Ethics Committee of the Faculty of Medicine – UnB (CAAE 93279318.9.0000.5558). All patients were included after signing the informed consent form. Our laboratory procedures are in full compliance with national and international regulations related to biosafety.

## Results

**Figure 2:**
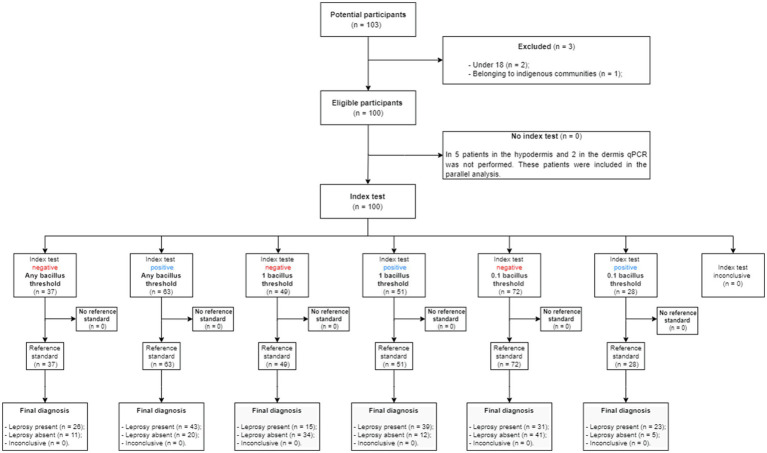
Study diagram of the flow of the participants during the study.

One hundred patients were included and divided into two groups: 54 were allocated to the leprosy group and 46 to the control group (Figure 2). In the control group, 20/46 (43.48%) patients were female, and 26/46 (56.52%) were male. In the leprosy group, 27/54 (50%) were female, and 27/54 (50%) were male (value of *p*=0.652). The mean age of the patients in the leprosy group was 47.9years, and that in the control group was 46.3years (value of *p*=0.580). The patients in the control group were finally diagnosed as follows: 20 with American tegumentary leishmaniasis, eight with cutaneous eczemas; six with cutaneous lupus; two with cutaneous lymphomas, one with squamous cell carcinoma, one with basal cell carcinoma; three with subcutaneous mycosis; two with cutaneous tuberculosis; one with fibroepithelioma of pinkus; one with atypical hidradenitis suppurativa; and one with syphilis.

Most of the leprosy cases (32/54, 59.26%) were characterized as paucibacillary (one primary neural leprosy, five tuberculoid, and 26 tuberculoid-borderline), while the others (22/54, 40.74%) were characterized as multibacillary (five borderline-borderline, three borderline-lepromatous, and 14 lepromatous-lepromatous). Among the total number of cases in the leprosy group, 23/54 patients (42.59%) showed leprosy relapse. We found no differences in the SSS results or skin or nasal swab quantification results between relapsed versus primary leprosy cases. No relationship between the total or relative number of sick household contacts was identified according to real-time PCR quantification (value of *p*: epidermis=0.540; upper dermis=0.625; inferior dermis=0.697; subcutaneous=0.645; nasal swab=0.663).

The superior and inferior dermis presented the greatest quantities of parasites, while nasal swab samples presented the lowest quantification results. However, the absolute (*p*=0.143) and relative quantification (*p*=0.132) results were not significantly different between different skin layers and swab samples (Tables 1–3). Almost all leprosy patients who tested positive according to nasal swab samples were classified as multibacillary cases, but three leprosy patients classified as paucibacillary cases had nasal swabs that were positive for *M. leprae* DNA but not for entire bacilli (Tables 2 and 3).

**Table 1:** Mean absolute and relative quantification results of the included samples.

	Absolute quantification	Relative quantification
	Mean bacillus number (standard deviation)	Mean bacillus number per milligrams of skin tissue (standard deviation)
Epidermis	260.59 (965.43)	80.04 (331.98)
Superior dermis	1260.89 (5246.18)	401.65 (1434.15)
Inferior dermis	1514.15 (7460.72)	661.04 (3491.63)
Hypodermis	820.96 (170.94)	2158.46 (10285.31)
Nasal swab	0.02 (0.06)	5.85 (22.66)

*Student’s t test: absolute quantification p=0.143; relative quantification p=0.132*.

**Table 2:** Sensitivity, specificity, and accuracy values considering different skin layers and real-time polymerase chain reaction thresholds.

Threshold	Sample	Sensitivity—positive/leprosy (95%CI)	Specificity—negative/control (95%CI)	Accuracy (95%CI)
Any bacillus threshold	Epidermis	57.41% – 31/54 (44.16–69.67)	76.09% – 35/46 (62.06–86.09)	66.00% (56.28–74.54)
	Superior dermis	59.26% – 32/54 (45.97–71.32)	73.91% – 34/46 (59.74–84.4)	66.00% (56.28–74.54)
	Inferior dermis	58.49% – 31/53 (45.09–70.74)	77.27% – 34/44 (63.01–87.16)	67.01% (57.16–75.56)
	Hypodermis	54.90% – 28/51 (41.38–67.73)	83.72% – 36/43 (70.03–91.88)	68.09% (58.11–76.64)
	Nasal swab	20.83% – 10/48 (11.73, 34.26)	100% – 28/28 (87.94, 100)	50.00% (39.03, 60.97)
0.1 bacillus threshold	Epidermis	38.89% – 21/54 (27.04–52.21)	89.13% – 41/46 (76.96–95.27)	62.00% (52.21–70.9)
	Superior dermis	40.74% – 22/54 (28.68–54.03)	86.96% – 40/46 (74.33–93.88)	62.00% (52.21–70.9)
	Inferior dermis	49.06% – 26/53 (36.12–62.12)	84.09% – 37/44 (70.63–92.07)	64.95% (55.05–73.71)
	Hypodermis	41.18% – 21/51 (28.75–54.83)	93.02% – 40/43 (81.39–97.6)	64.89% (54.83–73.78)
	Nasal swab	8.33% – 4/48 (3.288, 19.55)	100% – 28/28 (87.94, 100)	42.11% (31.65, 53.32)
1 bacillus threshold	Epidermis	24.07% – 13/54 (14.64–36.95)	100% – 46/46 (92.29–100)	59.00% (49.2–68.13)
	Superior dermis	33.33% – 18/54 (22.24–46.64)	93.48% – 43/46 (82.5–97.76)	61.00% (51.2–69.98)
	Inferior dermis	30.19% – 16/53 (19.52–43.54)	95.45% – 42/44 (84.86–98.74)	59.79% (49.84–69.00)
	Hypodermis	29.41% – 15/51 (18.71–43)	97.67% – 42/43 (87.94–99.59)	60.64% (50.53–69.91)
	Nasal swab	6.25% – 3/48 (2.148, 16.84)	100% – 28/28 (87.94, 100)	40.79% (30.44, 52.02)

*Analysis performed in the total population*.

**Table 3:** Sensitivity, specificity, and accuracy values associated with different skin layers and real-time polymerase chain reaction thresholds.

Threshold	Sample	Multibacillary			Paucibacillary		
		Sensitivity—positive/leprosy (95%CI)	Specificity—negative/control (95%CI)	Accuracy (95%CI)	Sensitivity—positive/leprosy (95%CI)	Specificity—negative/control (95%CI)	Accuracy (95%CI)
Any bacillus threshold	Epidermis	81.82% – 18/22 (61.48, 92.69)	76.09% – 35/46 (62.06, 86.09)	77.94% (66.74, 86.15)	40.63% - 13/32 (25.52, 57.74)	76.09% – 35/46 (62.06, 86.09)	61.54% (50.44, 71.55)
	Upper dermis	90.91% – 20/22 (72.18, 97.47)	73.91% – 34/46 (59.74, 84.4)	79.41% (68.36, 87.32)	37.50% – 12/32 (22.93, 54.75)	73.91% – 34/46 (59.74, 84.4)	58.97% (47.89, 69.22)
	Inferior dermis	90.48% – 19/21 (71.09, 97.35)	77.27% – 34/44 (63.01, 87.16)	81.54% (70.45, 89.11)	37.50% – 12/32 (22.93, 54.75)	77.27% – 34/44 (63.01, 87.16)	60.53% (49.29, 70.75)
	Hypodermis	80% – 16/20 (58.4, 91.93)	83.72% – 36/43 (70.03, 91.88)	79.25% (66.54, 88)	38.71% – 12/31 (23.73, 56.18)	83.72% – 36/43 (70.03, 91.88)	64.86% (53.5, 74.76)
	Nasal swab	36.84% – 7/19 (19.15, 58.96)	100% – 28/28 (87.94, 100)	74.47% (60.49, 84.75)	10.34% – 3/29 (3.581, 26.39)	100% – 28/28 (87.94, 100)	54.39% (41.59, 66.63)
0.1 bacillus threshold	Epidermis	68.18% – 15/22 (47.32, 83.64)	89.13% – 41/46 (76.96, 95.27)	82.35% (71.64, 89.61)	18.75% – 6/32 (8.889, 35.31)	89.13% – 41/46 (76.96, 95.27)	60.26% (49.16, 70.39)
	Upper dermis	72.73% – 16/22 (51.85, 86.85)	86.96% – 40/46 (74.33, 93.88)	82.35% (71.64, 89.61)	18.75% – 6/32 (8.889, 35.31)	86.96% – 40/46 (74.33, 93.88)	58.97% (47.89, 69.22)
	Inferior dermis	80.95% – 17/21 (60, 92.33)	84.09% – 37/44 (70.63, 92.07)	83.08% (72.18, 90.28)	28.13% – 9/32 (15.56, 45.37)	84.09% – 37/44 (70.63, 92.07)	60.53% (49.29, 70.75)
	Hypodermis	80% – 16/20 (58.4, 91.93^1^)	93.02% – 40/43 (81.39, 97.6)	88.89% (78.8, 94.51)	16.13% – 5/31 (7.093, 32.63)	93.02% – 40/43 (81.39, 97.6)	60.81% (49.42, 71.14)
	Nasal swab	21.05% – 4/19 (8.508, 43.33)	100% – 28/28 (87.94, 100)	68.09% (53.83, 79.6)	0.0% – 0/29 (0.0, 11.7)	100% – 28/28 (87.94, 100)	49.12% (36.62, 61.74)
1 bacillus threshold	Epidermis	45.45% – 10/22 (26.92, 65.34)	100% – 46/46 (92.29, 100)	82.35% (71.64, 89.61)	9.375% – 3/32 (3.24, 24.22)	100% – 46/46 (92.29, 100)	62.82% (51.73, 72.71)
	Upper dermis	68.18% – 15/22 (47.32, 83.64)	93.48% – 43/46 (82.5, 97.76)	85.29% (75, 91.81)	9.375% – 3/32 (3.24, 24.22)	93.48% – 43/46 (82.5, 97.76)	58.97% (47.89, 69.22)
	Inferior dermis	66.67% – 14/21 (45.37, 82.81)	95.45% – 42/44 (84.86, 98.74)	86.15% (75.73, 92.54)	6.25% – 2/32 (1.731, 20.15)	95.45% – 42/44 (84.86, 98.74)	57.89% (46.68, 68.35)
	Hypodermis	60% – 12/20 (38.66, 78.12)	97.67% – 42/43 (87.94, 99.59)	85.71% (75.03, 92.3)	9.677% – 3/31 (3.346, 24.9)	97.67% – 42/43 (87.94, 99.59)	60.81% (49.42, 71.14)
	Nasal swab	15.79% – 3/19 (5.52, 37.57)	100% – 28/28 (87.94, 100)	65.96% (51.67, 77.83)	0.0% – 0/29 (0.0, 11.7)	100% – 28/28 (87.94, 100)	49.12% (36.62, 61.74)

*Analysis performed in the subgroups of paucibacillary and multibacillary patients according to the Ridley and Jopling classification*.

**Figure 3:**
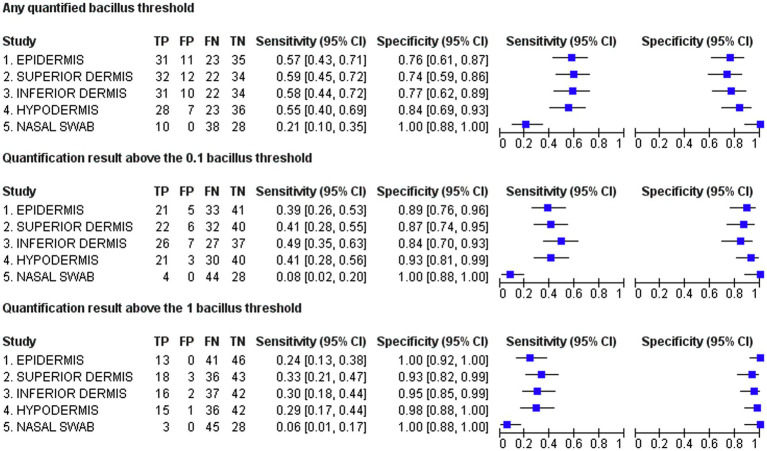
Forest plots representing sensitivity and specificity values. TP,true positive; FP,false positive; FN,false negative; TN,true negative.

**Figure 4:**
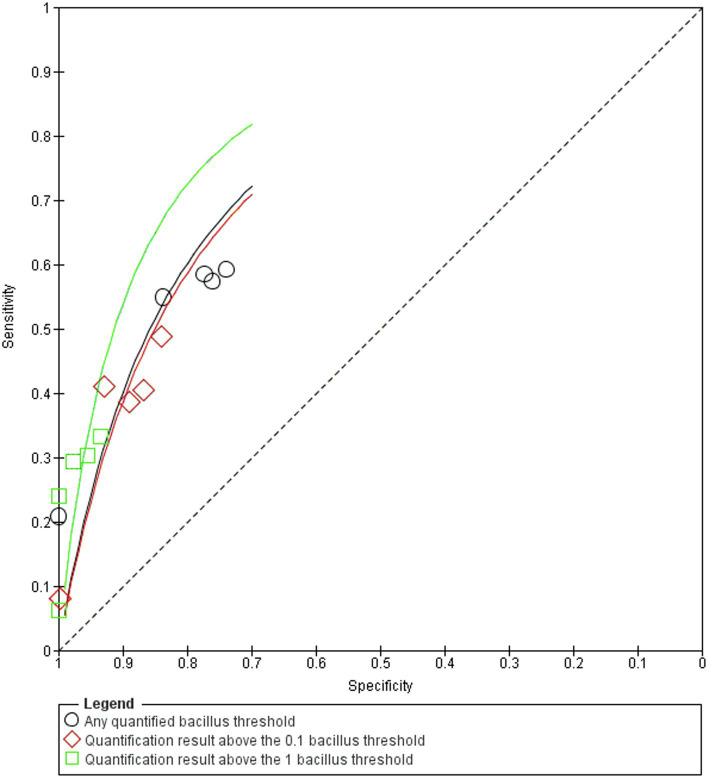
Summary receiver operating characteristic curve.

The best sensitivity of the real-time PCR test was obtained at the any quantifiable bacillus DNA threshold in the superior dermis [sensitivity=59.26% (95% CI=45.97–71.32)]. At the one bacillus threshold, the epidermis results reached 100% specificity (95% CI=92.29–100; Figure 3). The best accuracy for the hypodermis was found at the any quantifiable bacillus DNA threshold [accuracy=68.09% (95% CI=58.11–76.64)]. For swab samples, the best sensitivity of 20.83% (95% CI=11.73–34.26) was achieved at the any quantifiable bacillus DNA threshold. At all tested thresholds, 100% specificity was obtained for the swab samples. Following the R&J classification, sensitivity values were significantly higher in multibacillary patients, reaching 90.91% (95% CI=72.18–97.47) in the upper dermis at the any quantifiable bacillus DNA threshold, while the maximum sensitivity in paucibacillary cases was also found at the any quantifiable bacillus DNA threshold in the epidermis [sensitivity=40.63% (95% CI=25.52–57.74)]. Although real-time PCR positivity was greater in multibacillary patients, we found no direct correlation between molecular biology tests in any skin layer compared to SSS results (epidermis: *p*=0.256; superior dermis: *p*=0.136; inferior dermis: *p*=0.135; hypodermis: *p*=0.155; swab samples: *p*=0.085) probably because of the high number of paucibacillary patients who have, by definition negative SSS. In addition to the clinical forms of the leprosy patients, the SROC curve (Figure 4) showed that the main reason for any alteration of test accuracy was different thresholds. The different skin layers analyzed seemed to have no great influence on the accuracy values. Swab samples also showed significantly different accuracy properties than skin samples.

## Discussion

The diagnosis of leprosy is based on clinical findings, although the elucidation of atypical cases may require the use of complementary techniques (Gurung et al., 2019). In patients in the early stages of the disease, in whom the bacillary load is low, most existing examinations present limited sensitivity. The RLEP element is considered a sensitive and specific target for PCR (Martinez et al., 2011), although previous studies have not detected any influence of the chosen primer pair on the accuracy of the molecular diagnosis of leprosy (Gurung et al., 2019).

The *in vitro* properties of our designed primers and reaction were exceptionally efficient (Larionov et al., 2005). An eight-point quantification standard curve using cloned targets revealed satisfactory efficiency and a quantification limit of 0.03 bacilli. These results were considered an ideal starting point for a translational test of accuracy properties in real-life conditions. Although the dilution of the cloned control with human genomic DNA did not affect the *in vitro* properties of the test, we were aware that the clinical accuracy results of this technique had to be investigated in the target population. Many factors such as the presence of PCR inhibitors and a variable number of gene copies in individual pathogens may limit the results of quantification methods. As we agreed that the use of purified bacilli would also present limitations because of the natural limitation of microscopic methods, we designed a blinded, cross-sectional/cohort diagnostic accuracy study. According to methodological guidelines, these are essential measures to warrant the generalization of the performance of diagnostic tests in practice (Whiting et al., 2011).

The type of samples analyzed, consisting of different skin layers, did not influence the accuracy of the tested techniques. This result shows that although the separation of skin layers may not be extremely precise, this will have a limited influence on the PCR results. This result may be explained by the fact that modern extraction kits are efficient in isolating different sorts of biological samples (Manta et al., 2020; Abundo et al., 2021). Although there is controversy related to blood samples (Manta et al., 2020), previous studies have also not found significant differences in accuracy properties depending on different sample types when evaluating molecular biology techniques. The use of mucous samples collected using nasal swabs showed extremely limited results in relation to accuracy, even in multibacillary patients. These tests are not usually considered for diagnostic investigations, but their utility in monitoring aerial transmission has been examined (Araujo et al., 2016).

Our study did not find any biological markers of disease transmission. The absolute and relative numbers of bacilli found in any of the skin layers and in swab samples were not significantly related to the number of contaminated contacts. It is most likely that the sample size calculated for the test of diagnostic properties was not sufficient to test this assumption, which represents a limit of this study. This is especially true for swab samples, which we did not relate to the number of sick contacts, in contrast to the biological probability of the airborne transmission of leprosy, which has also been found in previous studies (Araujo et al., 2016). Interestingly, we found that three paucibacillary patients, classified using clinical criteria and SSS results, showed positive nasal swab samples at the any quantifiable bacillus DNA threshold. This result calls attention to the controversy related to the transmission of paucibacillary leprosy, which is still not well defined in the literature (Halder et al., 2001). Although we must consider that a considerable degree of subjectivity exists in leprosy classification and that multibacillary patients could be mistakenly classified as having paucibacillary forms, our results indicated that an entire quantifiable bacillus in the mucosa was not found in any of the paucibacillary patients. Biologically, it can be assumed that paucibacillary patients expel only DNA fragments of broken bacilli and not entire parasites. This finding disfavors the possibility of aerial transmission by paucibacillary patients.

The evaluation of the generated forest plots (Figure 3) showed that the application of different predefined thresholds clearly influenced the sensitivity and specificity values. This influence has been suspected in previous literature reviews but has never been systematically tested (Gurung et al., 2019). Threshold differences are exceedingly difficult to compare between studies, preventing analyses such as the evaluation of heterogeneity in meta-analyses (Hartzes and Morgan, 2019). Unless a clear standardization of molecular techniques is defined by the scientific community, this limitation is likely to persist. Multicentric studies, which are relatively uncommon for diagnostic techniques, might also be useful in overcoming this problem (Meisner et al., 2019).

The best sensitivity achieved was 59.26%, which was found at the any quantifiable bacillus DNA threshold in the superior dermis. At first glance, this might be considered a contradictory result according to the quantification limit of less than 1 bacillus. However, sensitivity limitations are expected to exist in clinical practice because of several factors, including human-related limitations and biological factors such as the presence of DNA inhibitors (Andrade et al., 2021). In line with our results, previous studies of leprosy reached a reduced summary sensitivity of approximately 75% for real-time PCR, proving that clinical conditions imply a variety of limiting factors in addition to those encountered under purely laboratory studies (Gurung et al., 2019). Furthermore, in most published articles, the evaluators were not blinded, and tests were conducted in controlled environments in which the diagnoses of cases and controls were previously known by investigators, representing so-called case–control accuracy studies (Gurung et al., 2019). This strategy naturally generates bias, artificially improving sensitivity and specificity (Whiting et al., 2011).

In the present study, the obtained specificity values were considerably more stable, with a minimal value of 73.91% for the superior dermis being obtained according to the any quantifiable bacillus threshold. The inverse proportionality of sensitivity versus specificity values (Figure 3) and the reduced diagnostic accuracy achieved when simulating real-life conditions reinforce the clinical relevance of the presented results. Our results clearly show that the results of the clinical application of diagnostic tests may be much different than what is found in initial laboratory tests, which is logically true not only for intervention clinical trials but also for the development of diagnostic techniques (Colli et al., 2014). In line with previous systematic reviews of the literature, our study shows that the application of molecular tests for the diagnosis of leprosy suffers from great heterogeneity if applied without well-established criteria. Most likely, the application of those tests in patients who have a greater chance of presenting multibacillary leprosy such as household contacts and patients with high levels of serum antibodies against phenolic glycolipid I is a promising strategy as most studies show that molecular tests have the best performance in this population. This strategy is also useful for breaking the transmission chain and must be pursued.

Recommendations for a good-quality SSS examination, used by the WHO as a leprosy case definition criterion, state that access to the deep dermis is preferable because *M. leprae* is particularly abundant in the lymphatic circulation characteristic of this location (Ministério da Saúde, 2010). Our quantification results support this assumption. Although the mean parasite load was greater in the superior and inferior dermis, *M. leprae* DNA was found in all skin layers, with no significant difference according to the depth of the evaluation. A previous study by Sevilha-Santos et al. (2018), who aimed to quantify the kDNA of *Leishmania* in the different layers of the skin, revealed a greater number of parasites in more superficial layers. The authors attributed the presence of DNA parasites in the epidermis to a probable host–defense mechanism known as transepidermal elimination (Goette, 1984). In line with previous studies that have used microscopy techniques (Okada et al., 1978; Seo et al., 1995; Satapathy and Kar, 2005), our study shows that this is probably an important mechanism of *M. leprae* depuration. However, the use of biopsies and smears that are too superficial may result in sensitivity loss. Future studies can compare the molecular quantification of skin layers to the microscopic count during histopathological examinations to generate confirmatory data. Immunohistochemistry is an interesting technique for this objective.

We can conclude that the type of skin sample collected for the molecular diagnosis of leprosy will have little influence on diagnostic accuracy, although superficial samples can result in sensitivity loss. In contrast, threshold changes greatly influence these properties. It is also evident that new diagnostic tools must be extensively tested in clinical practice to ensure their precise application in practice, as even highly efficient *in vitro*-tested techniques can suffer from a reduction in accuracy properties because of human and biological interference. Our results also show that paucibacillary patients may not be a relevant source of disease transmission.

## Data Availability Statement

The raw data supporting the conclusions of this article will be made available by the authors, without undue reservation.

## Ethics Statement

The studies involving human participants were reviewed and approved by Comité de Ética em Pesquisa da Faculdade de Medicina da Universidade de Brasília. Written informed consent to participate in this study was provided by the participants’ legal guardian/next of kin.

## Author Contributions

LS-S and SC contributed to formal analysis, investigation, and resources. CG contributed to conceptualization, data curation, formal analysis, funding acquisition, investigation, methodology, project administration, resources, software, supervision, validation, visualization, writing – original draft, and writing – review and editing. All authors contributed to the article and approved the submitted version.

## Funding

This study was financed by the Coordenação de Aperfeiçoamento de Pessoal de Nível Superior - Brasil (CAPES) - Finance Code 001.

## Conflict of Interest

The authors declare that the research was conducted in the absence of any commercial or financial relationships that could be construed as a potential conflict of interest.

## Publisher’s Note

All claims expressed in this article are solely those of the authors and do not necessarily represent those of their affiliated organizations, or those of the publisher, the editors and the reviewers. Any product that may be evaluated in this article, or claim that may be made by its manufacturer, is not guaranteed or endorsed by the publisher.
