# Supplementary material for: Standardization of SYBR Green-Based Real-Time PCR Through the Evaluation of Different Thresholds for Different Skin Layers: An Accuracy Study and Track of the Transmission Potential of Multibacillary and Paucibacillary Leprosy Patients
Source: Front Microbiol. 2021 Dec 7;12:758222. doi: 10.3389/fmicb.2021.758222 (PMC8753983; doi:10.3389/fmicb.2021.758222)
Supplement: Supplementary file 1 [file Table_1.DOCX]

**Supplementary File 1:**

1. Cloned sequence used as a positive control. Cloning was performed by Thermo Fisher Scientific GENEART (Regensburg, Germany)


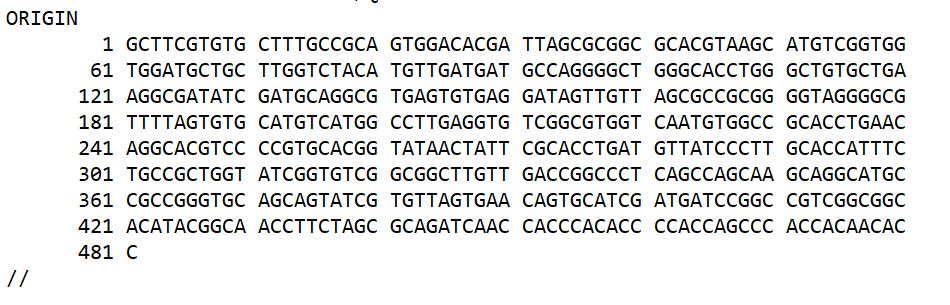


1. Standard curve generated at the Thermo Fisher Cloud (Thermo Fisher Scientific, Regensburg, Germany)

**
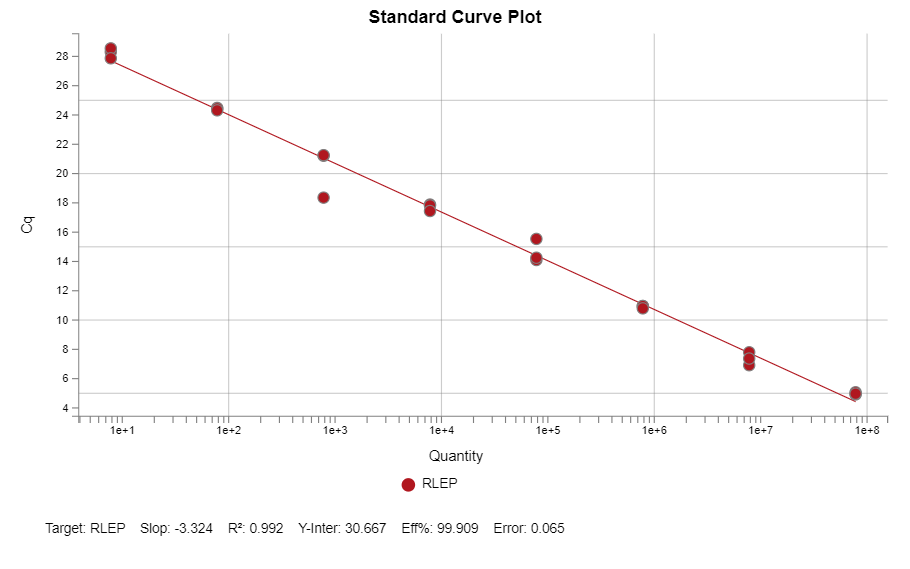
**
